# Approach and Avoidance During Routine Behavior and During Surprise in a Non-evaluative Task: Surprise Matters and So Does the Valence of the Surprising Event

**DOI:** 10.3389/fpsyg.2018.00826

**Published:** 2018-06-15

**Authors:** Achim Schützwohl

**Affiliations:** Department of Life Sciences, Brunel University London, Uxbridge, United Kingdom

**Keywords:** emotion, surprise, evolutionary psychology, action tendency, routine behavior

## Abstract

The hypothesis that emotions influence our behavior via emotional action tendencies is at the core of many emotion theories. According to a strong version of this hypothesis, these emotional action tendencies are immediate, automatic (unintentional), stimulus-based and directly linked with specific muscle movements. Recent evidence, however, provides little empirical support for this strong version during routine behavior, especially when the task does not require the evaluation of the stimuli. The present study tested the prediction that surprise interrupts routine behavior and triggers a threat avoidance response. In the presence of a threat-related stimulus, avoidance responses are relatively rapid, and approach responses impeded, even when the interrupted routine behavior is guided by a non-evaluative task goal. In contrast, approach and avoidance responses are predicted to be unaffected in the presence of a pleasant surprising stimulus. To test these predictions, in each trial the participants had to execute an approach or withdrawal movement depending on the location of a target stimulus. In the critical trial, either a picture of a pleasant or a threat-related animal was presented as target. Supporting the predictions, the initiation times for these movements were shorter in response to a threat-relevant than a pleasant surprising stimulus. Additionally, in the presence of a threat-related surprising stimulus, withdrawal movements were made faster than approach movements even though the participants performed a non-evaluative task. Implications and limitations of the present study are discussed.

## Introduction

A common assumption of evolutionary emotion theories is that emotions are linked to specific action tendencies (e.g., McDougall, 1908/1960; Lazarus, 1991; LeDoux, 1996). Specifically, these theories hypothesize that positively valenced stimuli trigger approach action tendencies whereas negatively valenced stimuli evoke avoidance action tendencies (e.g., Arnold, 1960; Bradley et al., 2001). These emotional action tendencies have presumably evolved because they facilitate the rapid approach to benign stimuli, and the rapid avoidance of harmful or threatening stimuli. According to a strong version of this hypothesis, these emotional action tendencies are immediate, automatic (unintentional), stimulus-based and directly linked with specific muscle movements (e.g., arm flexion is directly linked to affectively positive stimuli and arm extension directly linked to affectively negative stimuli; Solarz, 1960; Chen and Bargh, 1999).

However, two recent meta-analyses of studies that empirically tested this strong hypothesis came to the conclusion that it is currently not well supported (Phaf et al., 2014; Laham et al., 2015). Phaf et al. (2014) reported a significant compatibility effect between stimulus valence and approach and avoidance movements (i.e., faster approach responses in the presence of a positive than negative stimulus and faster avoidance responses in the presence of a negative than positive stimulus) only if the task required an explicit evaluation of the affective valence of the stimuli. However, in implicit tasks where the stimuli were to be categorized according to non-evaluative features such as the gender of faces shown or the grammatical status of words (e.g., Rotteveel and Phaf, 2004; Krieglmeyer and Deutsch, 2010; Krieglmeyer et al., 2010), no significant compatibility effect was found. Based on these results, Phaf et al. (2014, p. 14) concluded that the available data “argue against an immediate, unintentional, implicit, stimulus-based, and evolutionary-based or automatized link between affect and approach and avoidance.” It is important to note, however, that the lack of empirical support for the strong version of the hypothesis does not refute the assumption that emotional action tendencies have evolved to support the execution of approach and avoidance behavior.

Based on a larger sample of empirical studies due to more relaxed inclusion criteria Laham et al. (2015, p. 1082) found in their meta-analysis an “in all likelihood not larger than a small (compatibility) effect.” Although their meta-analysis revealed significant effects for both explicit and implicit evaluations tasks, consistent with Phaf et al. (2014), this small compatibility effect was even smaller for implicit than explicit evaluation tasks.

In addition, Laham et al. (2015) also found larger compatibility effects for human faces than for words or pictorial stimuli and for negative stimuli which were anger-related, which might indicate that these stimuli have higher ecological significance and are evolutionarily prepared to link to specific motor responses. Importantly, effect sizes were also significantly larger if the affective valence of a stimulus matched the explicit response labels that framed muscle movements as either positive or negative, irrespective of the type of muscle movement (e.g., arm flexion or extension). To illustrate, both flexion and extension movements in the presence of a positive stimulus were found to be made faster if these movements were positively framed as for example “toward,” “upward,” or “approach” than negatively as “away from,” downward,” or “avoid.”

This result obviously is not only incompatible with the strong version hypothesis of an automatic link between the affective valence of a stimulus and specific muscle movements (e.g., positive – flexion; negative – extension; Solarz, 1960; Chen and Bargh, 1999). It is also inconsistent with a second theoretical account of emotional action tendencies according to which these action tendencies regulate the distance between the affective stimulus and the actor (i.e., reduce the distance if the stimulus is positive and increase the distance if the stimulus is negative; e.g., Markman and Brendl, 2005) as flexion movements framed as positive in the presence of a positive stimulus actually increase the distance between the positive stimulus and the actor. Rather, this compatibility effect between the valence of the stimulus and the valence of the explicit response labels best supports a third theoretical account of emotional action tendencies, the evaluative coding account (Lavender and Hommel, 2007; Eder and Rothermund, 2008).

The evaluative coding account argues that both flexion and extension movements are facilitated in the presence of a positive stimulus if these movements are positively framed as “toward,” “upward,” or “approach” than negatively framed as “away from,” downward,” or “avoid” because of a match or overlap between the code representing the valence of the stimulus and the code representing the valence of the response label and this facilitative effect is assumed to be independent of distance regulation.

It is possible, however, that the lack of empirical support for the strong version of the evolutionary link between positive and negative affect and approach and avoidance responses, respectively, in the studies reviewed by Phaf et al. (2014) and Laham et al. (2015) was due to limitations of the research designs used, which were motivated by a too broadly conceptualized version of the emotion-action link. In the typical study, affective compatibility effects were tested (1) in a within-subjects design where the same participants responded with approach and avoidance to both positively and negatively valenced stimuli (2) during routine behavior (3) across a large number of rather uniform trials. In nearly all studies, furthermore, (4) the participants were either asked to indicate the affective valence of the stimuli, or to categorize the stimuli according to non-evaluative features such as the gender of faces shown, thus forcing participants to process and categorize certain inherent features of the stimuli (e.g., Rotteveel and Phaf, 2004; Krieglmeyer and Deutsch, 2010; Krieglmeyer et al., 2010). Moreover, (5) the negatively valenced stimuli used are typically not restricted to phylogenetically relevant objects with high ecological significance. Finally, (6) explicit response labels typically framed the movements as either positive or negative, allowing participants to employ a rather general mechanism of perception and action planning in the course of the experiment, that is not restricted to affective stimuli-response compatibility effects but also underlies non-affective stimulus-response compatibility effects based on for example the color, location or shape of stimuli (Theory of Event Coding: Hommel et al., 2001).

A more specific version of the evolutionary emotion-action hypothesis, however, may still be viable. This more restricted version predicts that automatic emotional action tendencies are elicited even if no evaluation or categorization of the stimuli is required in those cases where routine behavior is inappropriate to guide behavior. One such case is when the surprise mechanism gets activated. Thus, the present study takes a new theoretical and experimental approach to testing the automatic link between the affective valence of a stimulus and the ensuing approach or avoidance response based on a cognitive-evolutionary (CE) model of surprise developed by Meyer and colleagues (e.g., Meyer, 1988; Meyer et al., 1991, 1994, 1997; Schützwohl, 2009; Reisenzein et al., 2017). To achieve this goal, the present study examines approach and avoidance responses (1) in a between-subjects design while (2) a positive or negative unexpected stimulus interrupts routine behavior (3) in a single trial (4) using a task that does not require the analysis of any of the inherent features of the stimuli where (5) the negative stimulus is phylogenetically relevant and (6) in the absence of affective response labels.

## The Cognitive-Evolutionary (Ce) Model of Surprise

The CE model of surprise assumes that surprise is a (generally) adaptive, evolutionary-based mechanism that is elicited by discrepancies between current input and cognitive schemata. The surprise mechanism interrupts ongoing activities and enables and motivates processes that serve to analyze and cope with the schema-discrepant event, and, eventually, to remove the schema-input discrepancy (Meyer et al., 1994, 1997).

The present study is concerned with the evaluation of the schema-discrepant event’s implications for the individual’s well-being. In early versions of the CE surprise model, this evaluation was conceived of as an unbiased appraisal process determining whether the implications for the individual’s well-being are benign, negative/harmful or neutral. However, Schützwohl and Borgstedt (2005) argued that this appraisal process might not be as unbiased as previously thought. Instead, they proposed that the appraisal of well-being of the unexpected stimulus is geared to the rapid detection and preferential processing of possible impending harm or threat. This proposal was based on the assumptions that (a) surprising events were undoubtedly often dangerous during our evolutionary history (see already Darwin, 1872/1965, p. 222) and (b) the failure to quickly detect and preferentially process unexpected harm or threat presumably entails greater costs in the long run than the failure to quickly recognize unexpected benefits.

Support for the hypothesis that the appraisal of unexpected events prioritizes the detection of threat was obtained by Schützwohl and Borgstedt (2005; Study 2). In each trial of this experiment, two pictures were presented simultaneously. Depending on experimental condition, the participants had to decide whether at least one of the two pictures depicted either a pleasant or a threat-related stimulus. In a critical trial, pictures of both a pleasant and a threat-related animal appeared. These pictures were preceded by either a familiar or a novel, unexpected cue stimulus that elicited surprise. Response times indicated that during routine behavior (familiar stimulus), the pleasant animal was detected faster than the threat-related animal. In contrast, in the context of a surprising event, the threat-related animal was detected more reliably and faster than the pleasant animal. Additional empirical support for the hypothesis that the appraisal of unexpected event is biased toward the detection of threat was reported by Browning and Harmer (2012), who found that surprise increased the attention to potentially threatening faces. Finally, Topolinski and Strack (2015) recently reported that the immediate affect in surprise is negative which is also compatible with the assumption that the surprise mechanism prioritizes the detection of threatening stimuli.

The present study extends this line of research. This extension was based on the idea that the evolutionary strategy of coping with unexpected, potentially harmful events comprises not only the prioritized detection of threat but also an immediate avoidance response. This avoidance response is assumed to be independent of the affective valence and the task relevance of the schema-discrepant event and to facilitate withdrawal movements and to impede approach movements to threat-related stimuli. In support of this hypothesis, Herry et al. (2007) found that unpredictable stimuli induced avoidance behavior. Empirical support for this assumption can also be derived from the potentiation of the startle reflex (indicating an avoidance response) during unpredictable events (e.g., Grillon et al., 2006). In contrast, the detection that the surprising stimulus is neutral or benign indicates that an avoidance response would be inappropriate. I therefore predict that under these circumstances the prepared avoidance response is deactivated and thus has no effects on approach or withdrawal responses.

The objective of the present study is to provide an empirical test of the hypothesis that the surprise mechanism automatically triggers an avoidance response that is irrespective of the affective valence and the task relevance of the schema-discrepant stimulus. To test this assumption, a novel experimental procedure was developed: In each trial of the experiment, a target stimulus was presented either at the right or the left boarder of a computer screen. At the beginning of each trial, similar to Lavender and Hommel’s (2007) experimental set-up, the participants’ hand rested at the “home” plate, and the participants were asked to move it as quickly as possible to the left or right response plate depending on whether the stimulus appeared at the left or right boarder of the screen. For participants in the approach condition, the direction of the requested hand movement from the home plate to the response plates was toward the screen, which reduced the distance between them and the stimulus. The participants in the avoidance condition had to move their hand away from the screen, which increased the distance between them and the stimulus. This procedure allows separating the response times into an initiation time (the interval from the beginning of the target presentation until the participant lifts the hand from the home plate) and a movement time (the interval from the initiation of the response until the participant places the hand on the response plate).

To emphasize the non-evaluative nature of the task the targets in Trials 1 – 40 of the experimental group were lacking any affective valence as they consisted of bright squares presented against a black background. In contrast, in the critical Trial 41, a color photograph showing either a pleasant animal (a baby monkey) or a phylogenetically threat-related animal (a spider) appeared instead of the bright square. This stimulus change was intended to elicit surprise. In the control condition, the critical trial was identical to that in the experimental condition, but the presentation of the animal pictures was not surprising, because five pleasant and five threat-related animal pictures had already been presented during the preceding trials. These “animal picture trials” were interspersed with the bright square trials.

The following six predictions were tested: The presentation of the animal picture in the critical trial elicits surprise feelings in the experimental but not in the control condition. Initiation times in the critical trial are longer in the experimental than control condition, due to the interruption of ongoing activities caused by the surprise mechanism in the experimental condition. Initiation times in the critical trial are shorter in the presence of a surprising threat-related than a surprising pleasant stimulus, irrespective of the requested movement. In the presence of a surprising threat-related stimulus, the requested withdrawal movement times in the critical trial are shorter than the requested approach movement times. In the presence of a surprising pleasant stimulus, the requested withdrawal movement times in the critical trial do not differ from the requested approach movement times. Finally, both the initiation and movement times of the requested response in the control condition — i.e., during routine behavior, where the affective valence of the target stimuli is clearly task-irrelevant — are unaffected by the affective valence of the pictures in the critical trial.

## Materials and Methods

### Participants

The participants were 59 female and 40 male students. The female participants’ mean age was 24.8 years (*SD* = 4.9) and the male participants’ mean age was 24.6 years (*SD* = 3.0). They were recruited at the public places of the university and were not paid for their participation.

### Apparatus

**FIGURE 1:**
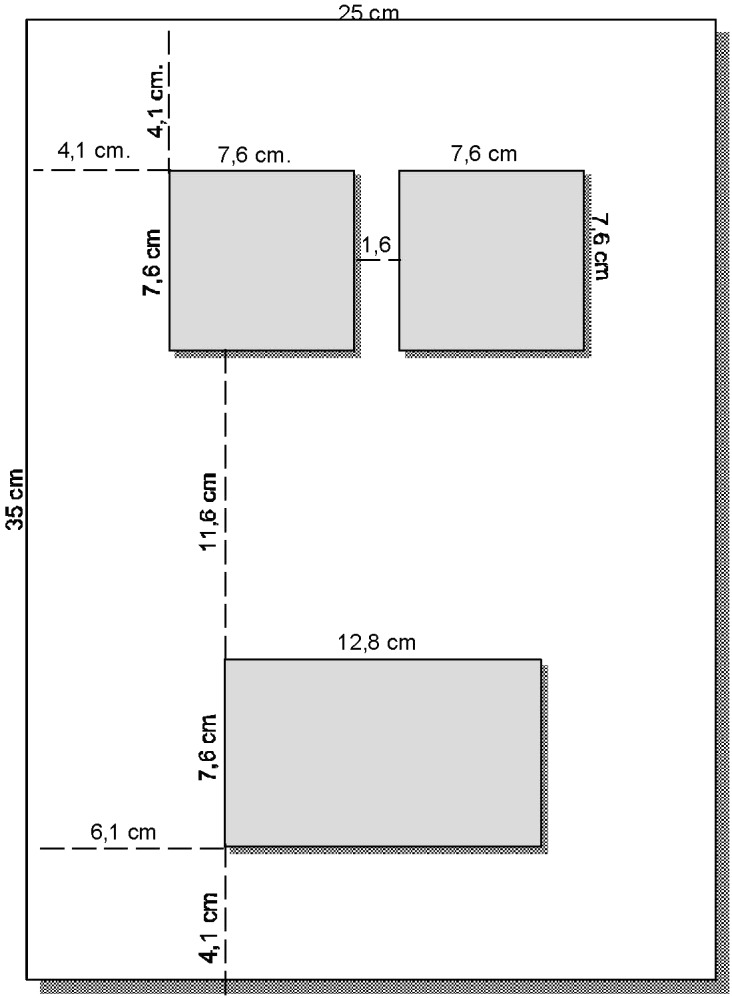
The response board.

The stimuli were presented on a 15” VGA monitor controlled by an IBM-compatible personal computer. The participants’ responses were recorded using three sensor plates that were mounted on a rectangular 35 cm × 25 cm plastic board (see **Figure 1**). The home plate was mounted at one end of the plastic board; its size was 12.8 cm × 7.6 cm. The two response plates were mounted side by side at the opposite end of the board; their size was 7.6 cm × 7.6 cm each. The distance between the two response plates was 1.6 cm and the distance between the home plate and a response plate was 11.6 cm. Reaction time (RT) measurement started with the presentation of the stimulus. When the participants’ hand left the home plate, an electric circuit was interrupted that caused the computer to register the initiation time. When the hand touched one of the response plates, another electric circuit was closed and the computer registered the movement time.

### Stimuli

Each trial started with the display of a bright arrow against a dark background for 150 ms at the center of the screen. The length of the arrow was 2.6 cm and it pointed either to the right or to the left. 400 ms after its disappearance, a target stimulus was presented either on the left or the right half of the screen. In the experimental condition, the target in Trials 1 – 40 was a bright square presented against a dark background. Four different sizes of squares measuring 0.5, 1, 2, and 4 cm, respectively, were used. In the control condition, five pleasant and five threat-related “animal trials” were randomly interspersed between 30 bright square trials during the first 40 trials. The pictures of the five pleasant animals showed a duckling, a hedgehog, a koala, a ground squirrel, and a penguin; the five threat-related animals shown were a scorpion, a bat, a snake, a hyena, and a wasp. In Trial 41, the critical trial, the target stimulus in both experimental conditions was either the picture of a pleasant baby monkey or of a threat-related spider. The color of both animals shown in the critical trial was of a similar brown. The size of the animal pictures was 8.0 cm × 7.7 cm.

**FIGURE 2:**
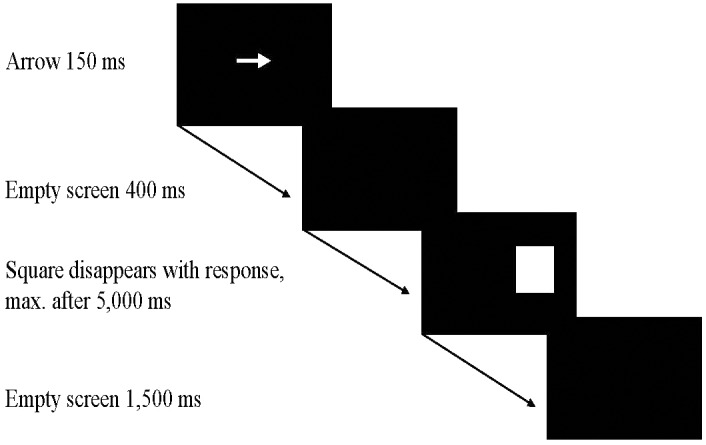
The sequence of events in a given trial.

In the experimental condition, each square size appeared equally often in Trials 1 – 40 in a fixed random order. In the control condition, the animal pictures always replaced the largest square. The center of the target stimulus was constant at 7.0 cm to the right or to the left of the center of the screen. The target stimulus disappeared when the hand was placed on one of the response plates or after 5,000 ms if no response had occurred until then. The next trial started 1,500 ms after the hand had returned to the home plate or – if no response had occurred within 5,000–1,500 ms after the target stimulus had disappeared. The sequence of events in a given trial is shown in **Figure 2**.

During Trials 1–40, the arrow was valid in 80% of the cases (i.e., it pointed at the correct location of the target stimulus). In the critical trial, the arrow was valid for half of the participants and invalid for the other half. Cue validity was introduced to prevent response initiation in response to the arrow, that is prior to the presentation of the target. In addition, the target in the critical trial appeared approximately equally often to the left and to the right of the screen center.

### Procedure and Instructions

The participants were tested individually in a dimly lit laboratory room. They were seated at a table approximately 50 cm from the computer monitor. The female experimenter remained in the room during the experiment so that she could answer questions that might come up during the experiment, especially in the critical trial in the experimental group. She sat at a table located behind the participant and oriented 90 degrees away, busying herself with some paper work.

The plastic board containing the sensor plates was located on the table approximately midway between the monitor and the participants. The experimenter provided verbal instructions describing the stimuli, the task and the validity of the arrow. Instructions were identical in the both movement conditions and only referred to the movement from the home plate to the response plate. Thus, no affective response labels were provided. The participants were instructed to place their right hand on the home plate and to move it as quickly as possible from the home plate to the response plates when the target stimulus appeared (depending on its location to the left or the right plate), and then to return the hand to the home plate. The participants in the control condition were additionally informed that in some trials, the target stimulus would be an animal picture. According to the event coding account (Hommel et al., 2001), action planning during routine behavior should be fairly easy because of the overlap of the location of the stimulus and the location of the response plate.

For participants with a requested approach reaction, the home and the response plates were arranged in such a way that the initial movement was toward the target; for participants with a requested withdrawal reaction, the initial movement was away from the target. This was accomplished by simply turning the plastic board by 180°. Thus, the approach movement started close to the participant and ended immediately in front of the screen, whereas the withdrawal movement started in front of the screen and ended close to the participant. Note that the instructions in the experimental condition referred to affectively neutral stimuli (bright squares of different sizes) and the requested movements were described without using any affectively valenced words, but only described the movements as from the home plate to the response plate, irrespective of the requested direction.

Ten practice trials were followed by 41 experimental trials. Immediately after the critical Trial 41, the participants were first asked whether they were surprised by the presentation of the animal picture in the last trial and, if yes, to indicate the intensity of their surprise feeling on an 11-point rating scale ranging from 0 (not at all surprised) to 10 (as surprised as one can possibly be).

### Design

The experimental design was a 2 (condition: experimental vs. control) × 2 (target valence: pleasant vs. threat-related) × 2 (requested movement: approach vs. withdrawal) × 2 (arrow validity: valid vs. invalid) between-subjects factorial design. The participants were randomly assigned to one of the 16 groups resulting from the combination of the four factors, with approximately twice as many participants assigned to the experimental than to the control condition.

## Results

### Surprise Feelings

As predicted, all but one participant in the experimental condition but only two control participants (one each in the pleasant and threat-related control group) reported that s/he was surprised by the presentation of the animal picture in the critical trial. Therefore, only the surprise ratings obtained from the experimental participants were analyzed further.

The mean surprise rating in the experimental condition was 7.06, indicating that the presentation of the target animal in the critical trial elicited considerable surprise. A two-way analysis of variance (ANOVA) of the surprise ratings, with target valence (pleasant vs. threat-related) and requested movement (approach vs. withdrawal) as between-subjects factors, revealed that the surprise ratings were influenced by the direction of the requested movement, *F*(1,64) = 7.36, *p* = 0.009, ${\text{η}}_{\text{p}}^{2}$ = 0.103. Inspection of the means revealed that participants requested to approach the target reported higher surprise (7.81) than those requested to withdraw from the target (6.39). The main effect of target valence and the interaction were not significant, *F*s < 1.

### Reaction Times

As mentioned earlier, the measurement procedure used in the present experiment allowed to separate the response times into an initiation time (the interval from the beginning of the target presentation until the participant lifted the hand from the home plate) and a movement time (the interval from the initiation of the response until the participant placed the hand on the response plate). These two RTs were separately analyzed. **Table 1** shows the mean initiation and movement times in the critical trials as a function of the condition, target valence and the requested movement.

**Table 1:** Mean initiation and movement times in milliseconds (SDs in parentheses) as a function of condition, target valence, and requested movement.

	Pleasant target		Threat-related target	
	Approach	Withdraw	Approach	Withdraw
**Experimental**				
Initiation	479 (183)	567 (389)	452 (273)	408 (224)
Movement	358 (184)	370 (229)	800 (629)	256 (134)
**Control**				
Initiation	309 (99)	248 (46)	297 (66)	263 (72)
Movement	211 (56)	196 (80)	184 (48)	241 (81)

#### Initiation Times in the Critical Trial

A four-way analysis of covariance (ANCOVA) of the initiation times in the critical trial with condition, target valence, requested movement and arrow validity (valid vs. invalid) as the between subjects factors and the baseline initiation time (the mean initiation time in the ten trials preceding the critical trial) as the covariate yielded a significant main effect of condition, *F*(1,82) = 19.81, *p* < 0.001, ${\text{η}}_{\text{p}}^{2}$ = 0.195. As predicted, initiation times were significantly longer in the experimental than in the control condition (477 ms vs. 279 ms). Although the predicted interaction between condition and target valence was not significant and thus was not as robustly supported by the data as it could have been, *F*(1,82), = 2.28, *p* = 0.135, ${\text{η}}_{\text{p}}^{2}$ = 0.027, the ANCOVA was repeated separately for the two conditions in order to directly test the prediction that the initiation times are shorter in the presence of a surprising threat-related than a surprising pleasant stimulus. Supporting this prediction, the main effect for valence was significant in the experimental condition, *F*(1,58) = 4.91, *p* = 0.031, ${\text{η}}_{\text{p}}^{2}$ = 0.078, reflecting shorter initiation times for the threat-related than the pleasant target stimulus (428 ms vs. 525 ms). In contrast, the same ANCOVA in the control condition revealed no significant main effect for valence, *F*(1,23) = 0.02. The remaining main and interaction effects were also not significant, *F*s (1,58) < 3.19, *p*s > 0.08, in the experimental condition, and *F*s (1,23) < 2.86, *p*s > 0.10, in the control condition. The remaining main and interaction effects of the initial ANCOVA were not significant, *F*s (1,82) < 1.90, *p*s > 0.17.

#### Movement Times in the Critical Trial

The movement times of two experimental participants (approach/threat-related and withdrawal/pleasant, respectively) exceeded their group mean movement times by more than three standard deviations. Their movement times were replaced by their respective group means (Ratcliff, 1993; Öhman et al., 2001). The four-way analysis of covariance (ANCOVA) of the movement times in the critical trial with condition, target valence, requested movement and arrow validity (valid vs. invalid) as the between subjects factors and the baseline movement times (the mean movement times in the ten trials preceding the critical trial) as the covariate yielded the predicted results. The main effect for condition was significant, *F*(1,82) = 11.94, *p* = 0.001, ${\text{η}}_{\text{p}}^{2}$ = 0.127, showing that movement times were longer in the experimental than in the control condition (433 ms vs. 208 ms). The main effect for the requested movement was marginally significant, *F*(1,82) = 3.76, *p* = 0.056, ${\text{η}}_{\text{p}}^{2}$ = 0.044. In addition, the significant two-way interaction between condition and requested movement, *F*(1,82) = 4.98, *p* = 0.028, ${\text{η}}_{\text{p}}^{2}$ = 0.057, as well as the marginally significant two-way interaction between target valence and requested movement, *F*(1,82) = 3.32, *p* = 0.072, ${\text{η}}_{\text{p}}^{2}$ = 0.039, were modified by a significant three-way interaction between condition, target valence and requested movement, *F*(1,90) = 6.30, *p* = 0.014, ${\text{η}}_{\text{p}}^{2}$ = 0.071. The remaining main and interaction effects were not reliable, *F*s < 2.

The significant three-way interaction was followed up with separate two-way between-subjects ANCOVAs for the experimental and control condition. For the control condition, this ANCOVA revealed no significant main effects, *F*s < 1, but an unpredicted marginally significant interaction between target valence and requested movement, *F*(1,23) = 4.26, *p* = 0.051, ${\text{η}}_{\text{p}}^{2}$ = 0.156, indicating that approach movements were slower than withdrawal movements for the pleasant target (211 ms vs. 196 ms) but faster than withdrawal movements for the threat-related target (184 ms vs. 241 ms). Subsequent *t*-tests, however, revealed no significant differences between any of the mean movement times involved in this interaction, *t*s < 1.72, *p*s > 0.10.

**FIGURE 3:**
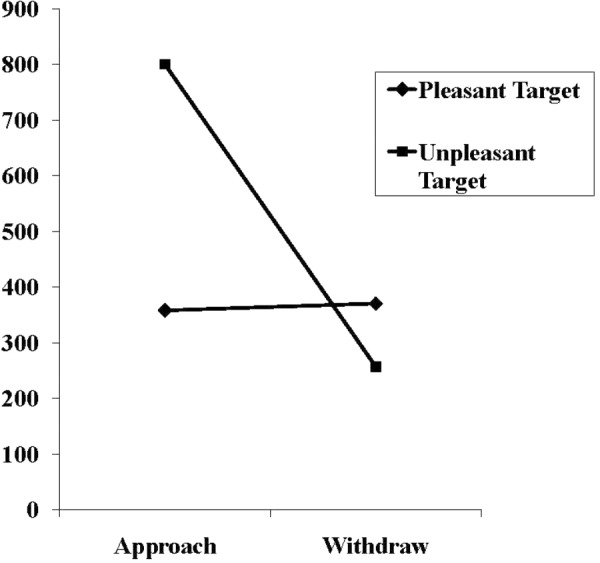
Mean movement times in milliseconds in the experimental and control condition for approach and withdrawal reactions in response to pleasant and threat-related targets.

In the experimental condition, the two-way interaction between target valence and requested movement was also significant, *F*(1,58) = 10.36, *p* = 0.001, ${\text{η}}_{\text{p}}^{2}$ = 0.152, thus modifying the significant main effects of the requested movement, *F*(1,58) = 9.55, *p* = 0.003, ${\text{η}}_{\text{p}}^{2}$ = 0.141, as well as the marginally significant main effect of target valence, *F*(1,58) = 3.78, *p* = 0.057, ${\text{η}}_{\text{p}}^{2}$ = 0.061. The mean movement times underlying this interaction are shown in **Figure 3**. As can be seen, the findings confirmed the prediction: The withdrawal movements were significantly faster than the approach movements in the presence of the threat-related target (257 ms vs. 800 ms), *t*(15, corrected) = -3.29, *p* = 0.005, *d* = 1.19. In contrast, withdrawal and approach movements times for the pleasant target did not significantly differ (370 ms vs. 358 ms), *t*(32) = 0.16.

Finally, the movement times were significantly longer in each of the experimental than their respective control groups, *t*s > 2, *p*s < 0.05, with the exception of the threat-related target with a requested withdrawal movement groups, *t* < 0.4.

## Discussion

The results of the experiment confirmed most of the predictions. As intended, the presentation of an animal picture in the critical trial elicited strong feelings of surprise in the experimental but not in the control condition. Additionally, the participants in the experimental condition requested to approach the target reported more intense surprise than those requested to withdraw from the target. A possible explanation of this finding is that the experience of a conflict between the requested movement, and the avoidance tendency triggered by the surprise mechanisms, may have contributed to the intensity of the surprise feeling by increasing the feeling of interference. Support for the hypothesis that the interference with ongoing activities can increase feelings of surprise has been found in several previous studies (e.g., Schützwohl and Krefting, 2001; Reisenzein and Studtmann, 2007; see Reisenzein et al., 2017).

Confirming the prediction, the initiation times in the critical trial were longer in the experimental than in the control condition, reflecting the interruption of ongoing activities caused by surprise. Additionally, the initiation times in the experimental condition were significantly shorter if the surprising stimulus was threat-related than if it was pleasant. However, the reader should keep in mind that the predicted interaction between condition and affective valence fell short of the conventional significance level and the present finding thus offers only qualified support for this particular prediction. Most importantly, withdrawal movements given a threat-related surprising stimulus were significantly faster than approach movements. In contrast, there were no significant differences between withdrawal and approach movements if the surprising stimulus was pleasant.

The only disagreement with predictions concerned the movement times in the control condition: Although, as predicted, the initiation times in the control condition did not differ as a function of the requested movement and the valence of the target, the approach movement time to a pleasant stimulus was somewhat longer, and that to a threat-related stimulus somewhat shorter, than the corresponding withdrawal movement times. As this pattern of findings for the control condition was not only unpredicted but is also hard to make sense of retrospectively, it might well be a spurious effect.

Taken together, the present findings in the experimental condition thus support the assumption of the CE surprise model, that the surprise mechanism interrupts ongoing activities, including routine behavior, and replaces them with a tendency to avoid threat. Furthermore, this was shown to occur even if the routine behavior was controlled by a non-evaluative task and in the absence of affective response labels: Irrespective of the requested movement, the initiation of a response was faster in the presence of a threat-related than a pleasant surprising event. Additionally, the execution of a withdrawal movement from a threat-related surprising event was much faster than the execution of an approach response toward this stimulus. In fact, the comparison of the movement times between the experimental groups and their respective control groups revealed that the withdrawal from a threat-related stimulus did not significantly differ between the experimental and control group, underscoring the urgency of the withdrawal from the threat-related surprising event. All other comparisons yielded longer movement times in the experimental groups. These significant differences in the movement times in the presence of a pleasant stimulus suggest that the response to a surprising pleasant event are considered as less urgent, thus allowing other processes to interfere with the execution of the requested response. These processes could concern the event analysis processes typically instigated by surprising events (Reisenzein et al., 2017). Alternatively, the interruption and resumption of the routine behavior in the surprise trial might result in a more voluntary and thus more time-consuming control of the execution of the requested movement, irrespective of its direction. In contrast, the substantially prolonged approach movement times in the presence of the threat-related surprising event presumably reflect the considerable efforts required to overcome the conflict between the requested movement and the withdrawal response automatically triggered by the surprise mechanism.

The present findings help to further refine the CE model of surprise proposed by Meyer et al. (1997) and Schützwohl (2000, 2009): The evidence from the present experiment suggests that the surprise mechanism not only prioritizes the processing of threat-related surprising events (Schützwohl and Borgstedt, 2005) but also prepares adaptive behavioral responses in the face of threat.

Because the preferential processing of threat-related information and the priming of a withdrawal response shares central characteristics with the fear mechanism (e.g., LeDoux, 1996), it seems possible that these responses are not part of the “surprise program” itself, but that surprise activates or recruits (parts of) the fear mechanism. This explanation, if correct, would not belittle the importance of the present findings, however. Rather, it fits well with Tooby and Cosmides’ (2008) evolutionary approach to emotions, which views emotions as superordinate programs that activate or recruit those psychological mechanism that in our ancestors’ past have contributed to the solution to specific adaptive problems. Unexpected harm or threat certainly constitutes an important adaptive problem. To solve this problem, it would be sensible for the surprise mechanisms to recruit an already existing mechanism specialized in coping with impending harm or threat. The activation of fear by surprise would also help explaining why the facial expression of surprise is similar to the facial expression of fear (e.g., Smith et al., 2005) and provides a theoretical account why the initial affect in surprise is negative (Topolinski and Strack, 2015).

What are the implications of the present data in the experimental condition for the three theoretical accounts of emotional action tendencies contrasted in the Introduction. The faster withdrawal than approach movement times in response to a surprising threat-related stimulus are incompatible with the evaluative coding account derived from the more general event coding account (Hommel et al., 2001) because this account applies only if both a negative stimulus and a negative response label are present. However, no affective response labels were provided. Instead the data provide initial empirical support of the strong version of an automatic, involuntary, stimulus-based direct link between a negative surprising stimulus and an avoidance response. As this avoidance response increases the distance between stimulus and actor, it is also compatible with the distance regulation account (e.g., Markman and Brendl, 2005). Further studies will be needed that try to disentangle the two theoretical accounts. The initiation and movements times in the control condition are mostly supportive of the event coding account: During routine behavior, the overlap between the location of the stimulus and the location of the response plates allows to use local codes for guiding behavior.

Certain limitations of the present study suggest directions for future research. In the present study, the approach movement consisted of an extension of the arm, which simultaneously resulted in a decrease of the distance to the target (Lavender and Hommel, 2007), whereas the avoidance movement consisted of a flexion of the arm which increased the distance to the target. These approach and avoidance responses were chosen in the present study because, as suggested by Schneirla (1959), they can probably be regarded as the default approach and avoidance responses in everyday life. Because of this choice of approach and avoidance responses, however, it is unclear which component of the response (muscular flexion or extension vs. increase vs. decrease of the distance to the target) was decisive. It would be highly interesting to know, however, whether the results obtained in the present study can be replicated with alternative operationalizations of approach and withdrawal movements such as the operationalization used by Rotteveel and Phaf (2004), who operationalized approach as arm flexion and avoidance as arm extension in the absence of a change of the spatial distance between the person and the affective stimuli. This would allow pinpointing more clearly the nature of the postulated, surprise-linked evolutionary avoidance tendency.

A second limitation of the present study is that only one pleasant and one threat-related stimulus was used in the critical trial. Although the two stimuli used (a baby monkey and a spider) can be considered prototypes of pleasant and threat-related stimuli, future studies should use additional target stimuli of each category to increase the generalizability of the findings. It should be noted, however, that previous studies that found evidence for the hypothesis of a threat detection and avoidance component of the surprise mechanism have already used a variety of pleasant and threat-related stimuli, including pictures of a squirrel and a ferocious dog (Schützwohl and Borgstedt, 2005; Study 2) as well as various animal words (Schützwohl and Borgstedt, 2005; Study 1).

Finally, future research should test the present predictions in real life encounters with pleasant and threat-related stimuli in the context of both routine behavior and a surprising event.

## Ethics Statement

The data for this study were collected in 1998 at the University of Bielefeld, Germany. At this time, there was no ethics committee in place and participants did not have to sign an informed consent form.

## Author Contributions

The author confirms being the sole contributor of this work and approved it for publication.

## Conflict of Interest Statement

The author declares that the research was conducted in the absence of any commercial or financial relationships that could be construed as a potential conflict of interest.
